# Towards a Sustainable Local Development of Instructional Material: An Impact Assessment of Locally Produced Videos on EFL Learners’ Skills and Individual Difference Factors

**DOI:** 10.3389/fpsyg.2020.02075

**Published:** 2020-09-09

**Authors:** Olusiji Lasekan, Margot Godoy

**Affiliations:** ^1^Departamento de Educación Media, Universidad Católica de Temuco, Temuco, Chile; ^2^Coordinación de Idiomas, Universidad de La Frontera, Temuco, Chile

**Keywords:** local videos, sustainable development, instructional material, English learning outcomes, Chile, individual difference factors

## Abstract

The purpose of this paper is to conceptualize the sustainability of local videos development, their integration in the EFL classroom, and their impact on students’ learning outcomes. The research aims as well to assess the impact of the videos on learners’ English language skills achievement and individual difference factors. We recruited a random sample of 100 and 76 students learning different levels of English in our English institute through an online survey. A cross-sectional study of perceived English language skill achievement and individual difference factors such as motivation, attitude, and cultural awareness was carried out. A combination of quantitative and qualitative approaches was used for data collection and analysis. Our conceptual framework showed that the sustainability of the local video production and its integration in the EFL classroom depends on the university’s funding policy, which is subjected to the positive impact of the videos on students’ learning outcomes. Also, the obtained results revealed that the videos fostered learners’ English language skills as well as their motivation to learn the language, positive attitudes toward its integration, and local cultural awareness. This work contributes to existing knowledge of sustainable local curriculum development by providing the first empirical evidence on the positive impact of sustainable local videos as an instructional material on students’ learning outcomes.

## Introduction

The importance of using videos to facilitate foreign language learning in classrooms cannot be overemphasized. A large and growing body of literature has investigated its positive impact on learners’ foreign language skills (Duffy, 2008; Almurashi, 2016). Besides language skills, video is considered to be a beneficial tool to simplify a difficult concept for learners and to capture their attention in the classroom (Moreno and Mayer, 1999). It is also viewed as an educational technology useful in promoting self-autonomous language learning (Hafner and Miller, 2011). Its incorporation into lesson plans has been proven to increase students’ involvement and participation in the classroom (Balcikanli, 2010). The author concludes that the pedagogical tool is capable of fostering students’ interest in the target language, which in turn reduces the stress that students face in the course of learning. Overall, it can be argued that video clips integration in EFL classroom activities can facilitate learning effectively.

Culture and language are interrelated (Rangriz and Harati, 2017). That is, to be able to learn and use the language correctly, an individual need to understand the culture of the target language (Mekheimer, 2011). Albeit, it is found that the local culture is more critical as the target language culture as well (Florentino, 2014). The argument is based on the need to reduce the foreign sense of a language so that the learner can culturally relate with it easily (Regmi, 2011). Thus, having such local cultural awareness assists learners to discover meanings in the target language by connecting the language with the context of their daily lives, including their personal, social, and cultural circumstances (Tavares and Cavalcanti, 1996). Numerous studies have shown that integrating local culture in the teaching and learning process enhances learners’ English language achievement (Choudhury, 2014; Haerazi et al., 2018). For that reason, Souza (2015) holds the view for applying the ecological perspective in foreign language teaching and learning. The theory is grounded on the notion that a student is an active agent of his or her environment, full of meanings, affordances, and signs (Van lier, 2006). Thus, employing an ecological perspective in EFL context will preserve or reclaim the learners’ local identity (Hornberger and Vaish, 2009) and enhance learners’ engagement (Han, 2019). Despite this, its integration in several EFL classrooms has been challenging. This ranges from teachers’ lack of understanding of the concepts of local culture (Wachdi, 1995) to the unavailability of local authentic materials to use in the classroom (Yuwono, 2005). The implication of this problem includes a negative attitude toward learning the language. In the case of students learning EFL in our English institute, students expressed their inability to comprehend and relate to foreign English textbooks that are showcasing only the British and American culture. A similar strong negative attitude toward the target language cultures has also been reported among Pakistani learners (Jabeen and Shah, 2011). Because of this, students’ motivation to learn the language is impeded, which consequently reduces the students’ retention rate in our programs. On the part of the institute’s management, using foreign instructional material and platform for teaching is not financially sustainable for us because of the high cost it incurs. This involves purchasing foreign licence of content for teaching, learning, and evaluation for the large number of students that the institute needs to teach every year. Considering that affordable price of instructional material for learners and institutions is an essential factor for successful integration of an EFL material into any curriculum (Richards, 1998), therefore, an ecological approach that can change the mindset of an institution into the creation of local socio-cultural content that has multiple positive influences on learners’ outcomes is needed (Souza, 2015).

Against this backdrop, our English institute decided to start producing and using local videos that express Chilean values and ethos for teaching content in 2014. We believe that the sustainable production of different videos over the years is a function of our ability to create a sustainable model that facilitate its production, integration in the classroom, and impact assessment on learning outcome. Since the inception of this project, the retention rate of the students has increased. In addition, the rating of the institute in terms of quality of teaching has improved. What is not known is the extent to which our videos foster learners’ motivation, cultural awareness, and language skills of the students. Thus, this paper aims to conceptualize the sustainability of local video development, integration, and impact assessment on learners’ motivation, attitude, cultural awareness, and language skills. The remaining part of this paper begins with reviewing the literature, construction of a conceptual framework, methodology, results, and discussion. Finally, the conclusion, which gives a summary and critique of the findings.

## Literature Review

Local development can be defined as a process of improving the economic, social, and environmental situation of an area based on the use of indigenous resources to improve its population well-being and quality of life (Dawkins, 2003). The most characteristic element of the concept is local resources (Coffey and Polese, 1984). This involve harnessing the economic and social dynamics of a particular territory so that its past and future are intrinsically linked to its use (Milán-García et al., 2019). The concept of local development was first integrated into sustainability in the 1970s (Amin, 1999). Sustainable local development (SLD) is defined as a form of development that has the capacity to sustain itself over time by respecting the constraints caused by a surrounding natural environment or an establishment (Mebratu, 1998). A body of works have investigated the feasibility of creating sustainable local development in different sectors, such as information communication technology in South Africa (Vosloo, 2005) and oil and gas sector in Nigeria (Heum et al., 2003). In the area of education, technology, and media applications have been employed as a form of sustainable local wisdom to develop curriculum for teaching and learning (Office of the National Education Commission, 1998). Pornpimon et al. (2014) explained that local wisdom could help in constructing cultural identity, which is critical for the indigenous people to sustain their livelihood. They further defined local wisdom as a kind of knowledge needed to promote national development efficiently. On this note, schools and community education are advised to adopt local resources and knowledge to contextualize a realistic education among learners. In recent years, numerous policy documents on the development and implementation of local curriculum have been issued in different countries such as Nepal (Subedi, 2018), New Zealand (Lockley, 2018), and Indonesia (Andrian et al., 2018). Concerning English language teaching, no EFL country has a robust policy on the teaching of English as a local content. Nevertheless, researchers understand the benefits of teaching an EFL in the local context (Haerazi et al., 2018). Emerging studies have focused on the level of the foreign and local cultural contents in English textbooks (Rashid and Ibrahim, 2018), teachers’ cultural preference in English textbooks (Sadeghi and Sepahi, 2018), and the impact of the local instructional material on students’ learning outcome (Choudhury, 2014). Although videos have been effective instructional material for language teaching (Duffy, 2008), far too little attention has been paid to its sustainable local production, its perception by the students, and its impact on students learning outcomes. Thus, the key research questions of this study are the following:

- What is the conceptual framework of sustainable local video production for English language teaching?
- What is the impact of the local videos on students’ English language skills achievement and individual difference factors such as motivation, attitude, and local cultural awareness?

## Conceptual Framework

As shown in Figure 3, a sustainable development model of our video productions and integration in EFL classroom was constructed. It is a model that provides a conceptual framework for the impact assessment on students’ learning outcomes. The greater detailed is described as follows:

### Institutional Funding

To provide quality, convenience, and flexible forms of education to students in our university, we institutionalize online learning programs and courses at the undergraduate and postgraduate levels. Every initiative and innovation, such as using video clips to facilitate learning in classrooms, is being promoted and funded. To sustain this initiative, the sponsorship model in which the institution assumes responsibility for funding is adopted. This covers incentives for both teachers and the production crews. The degree of annual funding for the content production of new videos is subject to their positive impact on learning outcomes. In sum, this project is supported using what might be called a producer-consumer model, where the support for an initiative is sustained through funding for production and distribution to a consuming population (Downes, 2007).

### Staffing

The decision to hire English teachers for the creation of video content is based on the new roles of twenty-first century teachers. The drive toward digital learning is a factor influencing the increasing number of global teacherpreneurs who are developing e-learning content and platforms, educational applications, and online marketplaces (Buckley and Nzembayie, 2016). Thus, the role of teachers has gone beyond face to face classroom teaching. Also, based on the demand of the students on the need to learn English that promotes Chilean culture and ethos, it is vital to hire Chilean English teacher that can reconcile both local context in video and English grammatical concept in the classroom. This is in line with the idea of using indigenous human capital and resources to foster development in every sector (Coffey and Polese, 1984).

## Content Production and Implementation

**Figure 1:**
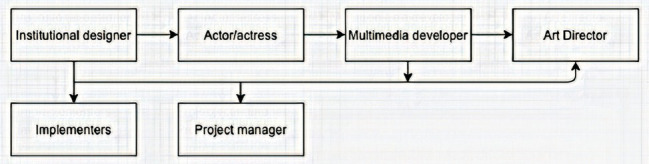
Coordination of content production team. Adapted from Parente (2010).

**Content production** is the process of developing and creating visual or written assets, such as videos or eBooks (Farmar, 2020). According to Parente (2010), the production team must consist of the following (Figure 1):

- **Instructional designers** develop the methodology and delivery systems for presenting course content. They formulate the objective of each video production.
- **Actors/presenters** are group of teachers who are cast and act out the scene of the video.
- **Multimedia developers** are group of professionals from the university’s multimedia unit. They are artists and designers who create interactive programs using a variety of graphic techniques to edit and produce the video.
- **Theater directors** are from the media department of the university. They participate in an artistic vision for a play, including selecting the cast, leading rehearsals, and monitoring the production’s pacing.
- **A project manager** is the coordinator of the institute is responsible for the successful initiation, planning, design, execution, monitoring, controlling, and closure of every production.
- **Implementers** are teachers who integrate the video into a classroom activity. One of the focal pedagogical approaches adopted by our institute is an active learning approach. It is an instructional strategy where students take an active role in the classroom by participating in learning activities and reflecting on their learning (Prince, 2004; Felder and Brent, 2009). Students are fully involved in the process of learning rather than being passive participants. This includes listening practices that help the students to absorb what they hear, short writing exercises in which students react to listening material, and complex group exercises in which students apply course material to situation of “real life,” and to new issues (Sandercock, 2015). Faust and Paulson (1998) stated that active learning takes different forms but generally involves four basic elements, which can occur individually in pairs or small or collaborative groups. The integration of these four elements (speaking and listening, writing, reading, and reflecting) makes active learning especially appropriate for interdisciplinary studies.

**Figure 2:**
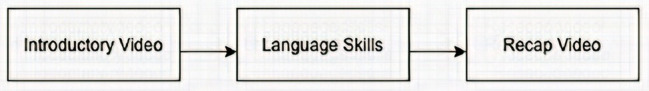
Integration of videos clips in classroom

Different concepts of English grammar and functional English are taught in numerous units of English course programs. Two video clips are created for two different purposes (Figure 2). The first video, which is called introductory video, is used as a guided lesson through the reading, speaking, listening, and writing tasks on the most important concepts being presented. In one of our numerous videos, the English grammar concept of “for,” “since,” and “used to” was taught through a video clip that compares the Chilean educational system and that of the United States. Immediately after that, a listening quiz on the video, reading, and speaking activity on the concepts was administered. The second video, which is called Recap video, is created to make students remember and reflect on what they have seen, read, written, spoken, and learned about the concept. This is carried out by recapping the first video and explains how the grammatical concept is connected to the first video.

**Figure 3:**
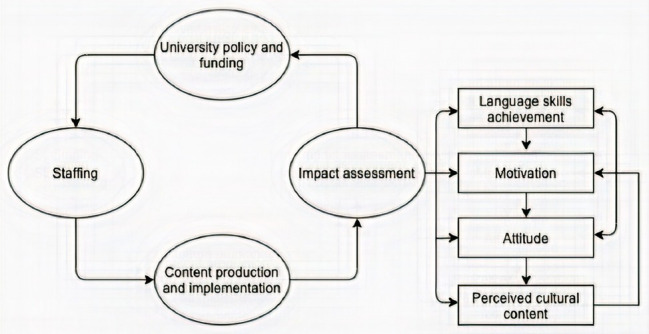
Conceptual model of sustainable local development for video production, its integration in EFL classroom and impact on students’ learning outcomes.

## Impact Assessment

Curriculum, instruction, and assessment are the three fundamental components of education (Orlando, 2011). Assessment, which is the most critical feature of good teaching, can be defined as the determination of students’ quality of their learning (Rowntree, 1987). Setting appropriate assessment tasks should involve questioning students in a way that demands evidence of understanding. It is also essential to use a variety of techniques to discover what students have learned (Jimaa, 2011).

Impact assessment is critical to the sustainability of our video production project. It affects educational decisions, policies, and funding of the project. Our impact assessments are monitored for quality during the videos production and when they are operational to determine that they are suitable for purpose and that they deliver their intended goals. For the purpose of evaluating the impact of the video on the students’ learning outcomes, it is important to evaluate its influence on learners’ individual difference factors. Individual difference is characterized as an enduring personal traits that are presumed to apply to everyone and on which people vary by degree (Dörnyei, 2014). Its role in foreign language learning has been used to explain different degrees of language achievement obtained by several groups of EFL learners (Lasekan, 2019). Thus, some of the key individual difference factors that could be considered for the impact assessment of the videos are as follows:

### Motivation

One of the most critical factors that determine the success of any individual in second language learning is the motivation (Dörnyei and Csizér, 1998). Winke (2005) also submitted that the ability of a learner to learn a L2 successfully is a function of whether he or she is motivated. Considering its positive impact on L2 learning, several authors have adopted different tools to stimulate motivation in EFL classrooms. This includes using extended music education (Nadera, 2015), ICT tools such as Webquest and Kahoot (Zorio, 2018), and video (Bajrami and Ismaili, 2016). The effectiveness of these tools has to lead to studies focusing on the motivational impact of these tools (Ferragut Martorell, 2018). In a study, which set out to determine the effectiveness of YouTube as a motivational tool among undergraduate students in Saudi Arabia; Alkathiri (2019) found that the tool is engaging and motivates them to participate in the class activities. Similarly, the usage of podcasting was perceived as a tool that fosters motivation in the EFL classroom (McMinn, 2008). The motivation stems from the students’ ability to hear a speech from a particular social group that they wish to learn about and perhaps identify with. No previous study has investigated the impact of locally developed videos on students’ motivation. This study provides an exciting opportunity to advance our knowledge of the relevance of local video on students’ motivation.

### Attitude

Attitude is defined as a way of thinking (the cognitive component), feeling (affective component), and intending to behave (the behavioral part) toward something (Rajecki, 1982, p. 33). Smit (1996) adds that language attitudes can be defined as strong positive or negative emotions experienced by people when they are faced with a choice between languages in a variety of situations or a variety of language learning. One’s attitude toward English, either positive or negative, will strongly influence his intention, orientation, effort, and the result of his or her learning (Hasbi, 2013). Karahan (2007) avers that “positive language attitudes let learners have a positive orientation towards learning English,” inferring that negative language attitude, in vice versa, will bear negative orientation. Studer and Konstantinidou (2015) established a strong relationship between students’ positive attitude and language achievement. Similarly, Hasbi (2013) compares students’ attitudes toward learning different English skills in EFL universities and English as a Second Language (ESL) universities. The study shows that those in EFL University have neutral-to-positive attitudes to listening, positive attitudes to speaking, positive attitudes to reading, and poor attitudes to writing. Also, the English students from ESL countries have better attitudes to reading (positive), and writing (neutral), and those from EFL country have better attitudes to speaking (neutral-to-positive) and listening (neutral-to-positive). The implications of such finding cause scholars to elicit for the opinion of learners on different authentic material so that they can identify those that foster a positive attitude toward learning (Hartatik and Rahmah, 2016; Halim et al., 2018). Example of positive attitude toward the usage of song lyrics, photography, television programs, songs, cartoons, picture stories, movies, and video in the classroom are reported by Sujono (2017). As regards students’ views on authentic video material, over 90% of participants believe that video is an excellent tool to facilitate the learning of English language skills with an emphasis on listening skills (Shahani et al., 2014). What is not yet clear is the student’s view of a locally developed video. It will be interesting to offers some valuable insights into a student’s perception of locally produced video.

### Cultural Awareness

The need to enculturate English in the EFL classroom is based on two posits. The first view states that “target language culture should be taught along with English to acculturate language learners into the culture of native English speaking countries (Byram and Fleming, 1998). The second perspective argues that target language culture should not be taught because English in most countries exists as an institutionalized variety (Kachru 1992). A study by Liu and Laohawiriyanon (2013) examined the perception of students on enculturation and acculturation in EFL classrooms has been conducted. The findings showed that learners were in favor of learning mostly about their own culture, followed by international target culture. In a study to identify different kinds of cultures in an English textbook, most of the participants identified more of native English-speaking culture in their textbook, and they considered the target culture to be essential and, thus, advocate for the inclusion of more of it in the curriculum (Xiao, 2010).

However, there has been little discussion about the perception and identification of culture in a locally developed video. It will be interesting to contribute to this growing area of research by investigating if the student can identify local ethos in the video.

### English Proficiency and Individual Difference Factors

Motivation, positive attitude, and cultural awareness in authentic material have been positive predictors of learners’ English language skills achievement. There is a plethora of studies that have given credence to this assertion. Rozmatovna (2020) performed an empirical study and found that both instrumental motivation and integrative motivation influenced learners’ language achievement. In another major study conducted among Vietnamese students showed a correlation between learners’ communication strategies and their attitude toward speaking of English (Bui and Intaraprasert, 2013). Finally, based on successful usage of local culture in texts to promote students’ comprehension of reading (Macalister, 2008), Nambiar et al. (2020) adopted reading modules focusing on local culture and content to foster learners reading, writing, listening, and speaking skills successfully. In sum, little is known about the relationship between’ English language achievement and motivational variable factors inspired by local videos.

## Materials and Methods

### Research Design

Intending to measure the effect of videos on EFL learning outcomes, previous studies have investigated their effect on learners’ individual difference factors such as motivation (Alkathiri, 2019) and attitude (Shahani et al., 2014). Thus, the purpose of this study is to assess the impact of our sustainable, locally produced video on students’ language achievements and individual factors such as motivation, attitude, and perceived cultural awareness. The findings could be valuable in establishing the importance of developing and using locally produced videos to boost learners’ English proficiency in EFL classrooms.

A questionnaire was adopted to obtain quantitative data required for analyzing respondents’ levels of perceived motivation, attitude, and cultural awareness. In addition, the relationships among them were investigated. Furthermore, interviews with selected participants provided qualitative data for further understanding of the variables for quantitative data. Employing two different instruments is necessary because interpretations that are built upon triangulation have a higher tendency to be stronger than those which rely on the more constructed framework of a single method (Lincoln and Denzin, 2000). Thus, this study used concurrent mixed methods to elicit quantitative and qualitative data. After the data collection, all the information was integrated and interpreted (Creswell, 2009, p. 14).

### Research Site, Population, and Sampling

This study adopted a random sampling technique in which a smaller sample size is culled from a larger population and uses it to research and make generalizations about the larger group (Banerjee and Chaudhury, 2010). Our choice was based on the simplicity of the technique (Taherdoost, 2016). However, to understand how different groups of participants view our videos so that their perspectives can be as representative as possible, we adopted maximum variation sampling (List, 2004). As shown in Table 1, all the informants (*n* = 176) are English learners recruited from different faculties of our university. It is a composition of students from the faculty of engineering, medicine, economics, and education studying different levels of English such as Elementary, Basic, Pre-intermediate, and Intermediate level of English.

**Table 1:** Percentage of sociodemographic characteristics of the study sample.

Variables	*n* (%)
**Gender**			
Male	77 (43.8)
Female	99 (56.3)
**Age**			
≤25	159 (90.3)
26–29	7 (4.0)
≥30	10 (5.7)
**Faculty**			
Agricultural and forestry sciences	14 (8.0%)
Legal and business sciences	29 (16.5%)
Social sciences	30 (17.0%)
Engineering	57 (32.4%)
Medicine	41 (23.3%)
Odontology	5 (2.8%)

### Research Instrument

#### Questionnaire

The instrument was used to collect quantitative data that would reveal the impact of locally produced video on students’ perceived language achievement, motivation, local cultural awareness, and different dimensions of attitude. The items in the questionnaire were drawn from various existing inventories designed to measure perceived language achievement and individual difference factors. By adopting face validity, we assumed that the instrument for this study is valid because each item addresses the specific and relevant aspects of perceived motivation, attitude, and cultural awareness (Holden, 2010). Moreover, a pilot questionnaire was administered to 12 participants. This was carried out to get their feedback on the accuracy, clarity, and cultural appropriateness of all items in the translated questionnaire. Items were validated based on errors of language equivalence noticed by Spanish-English translators and statistical experts. Some of the errors noticed by the participants while conducting pilot survey were inequivalence of content and constructs between the translated items and the original ones. The noted error was modified by a native speaker of Spanish and this was crossed checked by our statistical survey experts.

The questionnaire was divided into three sections. The first part of the questionnaire deals with respondents” demographic profile such as age, gender, and faculty of study in the university. While the second part consists of a 5-point Likert scale ranging from “strongly agree to strongly disagree” with eliciting respondents’ views on the variables, “very good to very poor” is used to measure the perceived quality of the videos and the third section is an open-ended question that elicits more responses regarding questions in Literature Review section.

To ascertain the perceived level of language skills achievement, we adopted the revised version of the instrument developed by Lialikhova (2014). It consists of (five items) perceived “listening,” “Speaking,” “Reading,” “Writing,” and “Pronunciation.” The perceived motivation was assessed by adapting four items developed by Willmot et al. (2012), Almurashi (2016), and Park and Jung (2016). With respect to awareness of local culture in the video, one item to evaluate cultural awareness was adopted (Yeganeh and Raeesi, 2015). Different dimensions of attitude consisting of five items were used to evaluate the perception of video effectiveness, usage, and relevance in a classroom (Shahani et al., 2014). Response scales consist of a 5-point frequency scale for all variables. Subscale scores are computed as simple averages and transformed into a 1–5 scale, with high values representing a high degree of perception and vice versa.

The interview section of the questionnaire emphasizes general views of the videos. A total number of five questions focus on how the material has helped the participants learn English, their view regarding the quality of the video in comparison to other free English teaching videos on YouTube, and how it has fostered their autonomous learning were probed. While most of the statements were adapted from different established studies on individual difference factors in second language acquisition, some were created to examine the themes that are yet to be investigated by previous works. The obtained data provided by the participants was analyzed using qualitative thematic analysis to answer the questions and accomplish the objective of the present study. Our data was manually-coded. We adopted inductive coding. This involves categorizing opinions into common themes (Cooper et al., 2012). The four themes that emerged were used to address issues related to the core objectives under investigation.

## Data Collection and Analysis

Data for this study were retrospectively collected through an anonymous online survey. The data was administered at the end of the English courses during the academic year 2018/2019. This is to ensure that all of the participants are familiar with the videos to provide broad perspectives and assessment of the videos. All research ethics codes for data collection, which involves explaining the objective of the study to the informants, informing of the right to decline to participate, and explaining the low potential risk involved by providing the greatest sense of respondent confidentiality were upheld.

Collected data were analyzed using SPSS v25.0. The students’ demographic distribution was obtained through the analysis of the proportion of students in different faculties within the university (Table 1). The overall mean score of perceived language skill, motivation, and attitude was assessed. The total validity was tested by calculating the reliability of the items adopted in the instrument through Cronbach’s alpha. Finally, a correlational analysis was carried out to gain insights into the relationship between the perceived individual difference factors and perceived English language skills achievement.

## Results

The sociodemographic characteristics of the participants are summarized in Table 1. The sample includes a higher proportion of female students (56.3%) than male students (43.8%). The majority of the informants are below 25 years old (90.3%). The highest percentage of the respondents are studying in the faculty of engineering (32.4%), followed by students in faculty of medicine (23.3%), then by faculty of social sciences (17%), and finally by students of faculty of legal and business sciences (16.5%).

The impact of the videos on students’ perception of their English language skills achievement and the level of reliability of the items adopted for the measurement are highlighted in Table 2. Taking into consideration that the higher the mean value, the higher the positive perception, and vice versa. From the table, it can be seen that a large proportion of proportion agree that the locally developed videos boost their overall language skills (3.85). Out of all the skills, the majority of them claim that the video fosters their listening skills (4.22). Also, the overall internal consistency of items at 0.913 can be considered to be acceptable (Taber, 2018).

**Table 2:** Mean value of perceived english language skills achievement.

Language skills	Items	Mean	SD	Min	Max	Cronbach’s alpha
Reading	1	3.87	1.01	1	5	0.890
Listening	1	4.22	0.86	1	5	0.904
Writing	1	3.52	1.17	1	5	0.898
Speaking	1	3.85	1.02	1	5	0.883
Pronunciation	1	3.84	1.05	1	5	0.888
Language skill total scale	5	3.85	0.88	1	5	0.913

Table 3 demonstrates the effect of the videos on students’ perceived motivation to learn. It can be seen that the majority of students agree that the locally developed video enhanced their motivation in the classroom (3.79). A large number of respondents (4.18) assume the video is more motivating than PowerPoint presentations. Moreover, the overall reliability of the items is acceptable at 0.878 (Taber, 2018).

**Table 3:** Mean value of motivation caused by the locally developed video.

Items	Number of items	Mean	SD	Min	Max	Cronbach’s alpha
Watching the institute educational videos in class is more motivating than watching a PowerPoint presentation	1	4.18	1.02	1	5	0.870
The videos capture my attention better than any other English educational video	1	3.79	0.99	1	5	0.815
The videos motivated me in classes to learn English	1	3.69	1.03	1	5	0.823
The videos of the class motivated me to see more videos on the platform	1	3.49	1.06	1	5	0.863
Motivation total scale	4	3.79	0.87	1	5	0.878

From the data in Table 4, participants’ express different dimensions of attitude toward the locally developed video. A great deal of them have a strong positive attitude toward its integration into classroom activities and recommendations to teachers, a considerable number of students assume the video is relevant to the course content (4.16). Furthermore, the overall internal consistency which stands at 0.942 can be considered to be acceptable for assessment (Taber, 2018).

**Table 4:** Mean value of attitude toward the locally developed video.

Items	Number of items	Mean	SD	Min	Max	Cronbach’s alpha
The videos facilitate the learning of the English language	1	4.14	0.91	1	5	0.926
If I were a teacher, I would use the institute YouTube channel	1	3.91	1.05	1	5	0.937
The videos enable me to better understand the English language	1	4.06	0.91	1	5	0.928
The videos help me to achieve the assigned tasks in English rather than working with texts	1	3.83	1.06	1	5	0.931
The videos used on the platform were relevant to the course content	1	4.16	0.96	1	5	0.931
The videos used in classes increased my participation in the classroom	1	3.80	1.01	1	5	0.937
Attitude total scale	6	3.97	0.85	1	5	0.942

Table 5 demonstrates students’ perceived level of Chilean cultural values and ethos in the videos. Only one item is used to assess this dimension. A substantial number of respondent agree that the video promotes Chilean culture than foreign ones.

**Table 5:** Mean value of perceived local cultural awareness.

Items	Number of items	Mean	SD	Min	Max
The videos promote Chilean culture more than foreign ones	1	3.66	1.04	1	5

Table 6 provides the level of correlation between variables and that of language skills. A strong positive correlation (<0.001) is observed between each variable and the language achievement skill. That is, each variable influence positively the language skills development of the students.

**Table 6:** Degree of correlation between each variable and total language skill.

Variables	Language skill	Degree of correlation
Individual difference factors	M (SD)	*p*
Perceived fotivation	0.773	<0.001
Perceived attitude	0.854	<0.001
Perceived cultural awareness	0.610	<0.001

*M, Mean; SD, Standard deviation*. ^*^p < 0.05; ^**^p < 0.01; and ^***^p < 0.001. (Schober et al., 2018).

In the open-ended questions of the instrument. Participants were asked to express their opinions regarding the perception of the videos. Some of the main themes that emerge from their responses are the following:

### Quality of Production

The vast majority of the interviewees commented on the quality of the video. Some describe the quality in terms of audio and visual quality. As one interviewee said “*The videos are more visually organized and pleasing to the eye. They are good audiovisual material to learn English better*.” Some believe the quality is enhanced because it is well subtitled with English, which helps them in their writing production. For example, one interviewee said “*the subtitles that the videos have, you can know how these words are written*.”

### Clarity of Content

Some participants believed that the videos help in facilitating their understanding of every grammatical concept of each moodle and good relationship with the teachers. That is, it helps them to understand the context at which certain grammatical concepts should be used. For example, one interviewee said “*Watching the English videos helped me better understand each unit’s topics*.” Another common view among interviewees was that it is easier to understand the concept being taught because their teachers are also the main actors or actresses in the video clips. As one participant mentioned “*those who make the videos are the institute teachers, and as this is the case, it allows us to have a closer relationship with them in class*.”

### Language Skills

A significant number of the participants expressed how the video enhanced their language skills in reading speaking, listening, and writing. Out of all the skills, listening skill is mostly emphasized for its enhancement by the videos. One of the comments encapsulates this perspective “*They have helped me a lot with the listening part*.”

### Cultural Content

The participants provided more information on the perceived Chilean cultural ethos in the video. They appreciate that the video promotes local culture rather than American or British culture. One participant commented that the videos focus on promoting Chilean culture and society, in contrast to other English videos, which only focus on English or American. Some appreciate the fact that the videos are shot in the campus. Therefore, promoting campus life and issues. As one respondent indicated “*the videos generate university’s identity; they should make them more massive, such as educating in the casino with short explanatory videos and relating them to important topics such as gender, sexuality, security, academic and administrative processes etc*.”

## Discussion

In reviewing the literature, no data was found on the development of sustainable production of local videos and its impact on foreign language learning outcomes. Thus, this study set out with the aim of assessing the impact of our sustainable, locally produced video on EFL learners’ language achievements and individual factors such as motivation, attitude, and perceived cultural awareness.

To address research question 1, we conceptualized the sustainable model of video production and its integration in an EFL classroom. This is followed by assessing its impact on students’ motivation to learn, attitudinal dispositions toward learning, awareness of the local cultural content, and perceived language skills achievement. A model whereby the university’s policy and funding promote the hiring of English teachers to produce video clips for English learning was constructed. The sustainability of the production model depends on continuous funding of the video, which is subjected to its positive impact on students learning outcomes.

In relation to language skills, a sizeable number of the respondents believed that the video fostered their reading, listening, speaking, pronunciation, and writing skills. These results match those observed in an earlier study conducted in France (Duffy, 2008). Listening is considered to be the most enhanced skill by the video. This view is also supported by previous research on the stronger connection between video utilization in classroom and listening skills (Shahani et al., 2014).

Another important finding was that majority agreed that the videos motivate them to learn the language. These findings further support the idea of using video to enhance motivation in the EFL classroom (Dörnyei and Csizér, 1998). A possible explanation for this might be their higher preference for visual rather than PowerPoint presentation.

To assess the level of awareness of local cultural ethos and value in the content, respondents were asked to rate the level of Chilean ethos compare to the foreign one. The most interesting finding was that extensive number of the respondents admit that the videos express more of Chilean ethos than the foreign ones. Though no work has been done on perception of cultural awareness of a locally produced video, but the finding is in line with a study that investigates the perception of cultural awareness in English textbooks (Xiao, 2010). These data must be interpreted with caution because only one item is used to assess this dimension. Further work is required to create a robust instrument to measure local cultural awareness in locally produced videos.

To determine the factors that influence English language skills, correlational analysis between the several motivational variables and the language skills was carried out. It was found that perceived cultural awareness, motivation, and positive attitudes toward the video are highly correlated with the perceived language skills. That indicates that the fostering of language skills can be linked to the individual difference factors induced by the videos. This also accords with our earlier observations on the relationship between enhanced English proficiency and motivation (Rozmatovna, 2020), positive attitude (Bui and Intaraprasert, 2013), and perceived local cultural awareness (Yeganeh and Raeesi, 2015). This combination of findings provides some support for the positive impact of the conceptual sustainable local video production on students’ English language learning outcomes.

The present results are significant because it helps us to understand that the higher retention rate and perceived good quality of teaching of our institute could be linked to the positive impact of locally developed video integrated on students learning outcomes. The implication is that we have used local resources and human capital to improve the quality of teaching in our institute. This has justified the importance of adopting local content to foster any form of development at a national level (Pornpimon et al., 2014). Furthermore, developing local material for teaching is more cost-effective because we do not need to depend on foreign teaching and evaluative materials, which are exorbitant. Consequently, we have been able to save financial resources because we have not been purchasing foreign teaching materials. This is another advantage of using local content in terms of saving costs (Heum et al., 2003). Our justification for adopting this approach is strengthened because it allows us to expand and develop the artistic and multimedia skills of our teachers. This is critical for their in-service professional development as they are learning their new multi-task roles as twenty-first century teachers (Buckley and Nzembayie, 2016). This is another successful attempt to use local content to develop human capital in the area of education.

Finally, the empirical findings in this study provide a new understanding of the role and significance of sustainable local development, individual difference factors, and ecological perspective in the production of local video instructional material. Our sustainable local development framework has demonstrated the process of how teachers can key into their institution’s local content policy, collaborate, develop instructional material, and then integrate it into their classrooms. Our study has also responded to a scholar’s suggestion to apply the ecological perspective theory in EFL learning (Van Lier, 2010). This involves employing learners’ local ethos to foster the target language learning process (Nambiar et al., 2020) while maintaining or reclaiming their local identity (Hornberger and Vaish, 2009). The impact of the application is evident in the participants’ views regarding the quality of our videos and its local cultural appropriateness. Concerning the impact assessment of our videos on the students’ language achievement, individual difference factors have been proven in our study to be a strong predictor of foreign language learning. For example, the positive correlation among these factors and their significant language achievement is a proof that the instructional videos are of high quality in cognitive load and promotion of active learning.

## Conclusion

The main goal of the current study was to conceptualize the sustainability of local video development, integration, impact assessment on learners’ motivation, cultural awareness, and language skills. One of the most significant findings is that the videos are perceived to foster learners reading, listening, writing, and speaking skills. Additionally, students expressed a positive impact on their motivation to learn the language. Also, participants’ express different dimensions of attitude toward the integration of the video in the classroom and the learning of the language. Moreover, most of the participants believe our videos promote more of Chilean cultural values and ethos than foreign ones. Finally, correlation analysis revealed a strong positive relationship between cultural awareness, positive attitude, and motivation with learners’ language skill perceived achievements. As an institution, the current findings make us understand the factors responsible for the excellent performance of our students. The impact assessment instrument is also effective in evaluating the quality of our videos periodically, which is critical to the sustainability of video funding, production, and integration in the classroom. Concerning research contribution, this the first study shedding light on how to construct sustainable local wisdom and resources to produce local instructional material for English language teaching. The findings in this report are subject to at least two limitations. First, the adoption of one item scale to measure the construct of cultural awareness causes its low content validity. The second limitation lies in the fact that this study used a self-perception survey to assess the language skill achievement of the learners. This can compromise the validity of their responses due to self-flattery (Oller and Perkins, 1978). This limitation means that study findings need to be interpreted cautiously. Thus, a correlation analysis between student’s performance in English skill exams and their views on individual difference factors could provide more definitive evidence on the impact of the variables on the students’ language achievement. Regarding cultural awareness, more robust items need to be created to investigate the level of local cultural content in our videos. By and large, this study can be used as a template to develop a sustainable approach for creating local instructional material for English language teaching in EFL classrooms.

## Data Availability Statement

The raw data supporting the conclusions of this article will be made available by the authors, without undue reservation.

## Ethics Statement

Ethical review and approval was not required for the study on human participants in accordance with the local legislation and institutional requirements. Written informed consent to participate in this study was provided by the participants’. All research ethics codes for data collection, which involves explaining the objective of the study to the informants, informing of the right to decline to participate, and explaining the low potential risk involved by providing the greatest sense of respondent confidentiality were upheld.

## Author Contributions

All authors listed have made a substantial, direct and intellectual contribution to the work, and approved it for publication.

### Conflict of Interest

The authors declare that the research was conducted in the absence of any commercial or financial relationships that could be construed as a potential conflict of interest.
